# Manganese and its Role in Biological Processes

**DOI:** 10.1155/MBD.2000.167

**Published:** 2000


					Metal-Based DJ'ttgs I/oi. 7, No. 3, 2000
MANGANESE AND ITS ROLE IN BIOLOGICAL
PROCESSES
volume 37 of the series
"METAL IONS IN BIOLOGICAL SYSTEMS"
edited by Astrid Sigel and Helmut Sigel
Marcel Dekker, Inc.
Volume 37, devoted to manganese, an essential element for many living systems, including
mammals, with more than 20 identified functions in enzymes and proteins, describes its role in
biology, offers a comprehensive and timely account of this fascinating topic by an impressive
collection of distinguished international authorities:
Gerald T. Babcock and Curtis W. Hoganson (USA), Ralf Bogumil, Reinhard Kappl and
J6rgen HLitterrnann (Germany), David W. Christianson, David E. Ash and J. David
Cox (USA), James D. Crowley, Deborah A. Traynor and David C. Weatherburn (New
Zealand), Valeria Cizewski Culotta (USA), Richard J. Debus (USA), Jodi L. Ensunsa
(USA), Andrew L. Feig (USA), Michael H. Gold, Heather L. Youngs and Maarten D.
Sollewijn Gelpke (USA) Stephen E. Halford, Geoffrey S. Baldwin and Niall A.
Gorrnley (UK), A. Joseph Kalb (Gilboa), Jarjis Habash, Nicola S. Hunter, Helen J.
Price, James Raftery and John R. Helliwell (Israel/UK), Carl L. Keen Jodi L. Ensunsa
and Michael S. Clegg (USA), James C. K. Lai, Margaret J. Minski, Alex W. K. Chan
and Louis Lim (UK), James J. Morgan (USA), Vincent L. Pecoraro and Wen-Yuan
Hsieh (USA), James E. Penner-Hahn, Jungwon Hwang and Derek W. Yoder (USA),
Lawrence Que, Jr. and Mark F. Reynolds (USA), George H. Reed and Russell R
Poyner (USA), Zdenko Rengel (Australia), Frank Rusnak (USA), (USA), and James W.
Whittaker (USA).
In twenty stimulating chapters, "Manganese and Its Role in Biological Processes" highlights first the
availability of this element to organisms, its uptake and transport in microorganisms and plants as
well as its role in health and disease of animals and humans including its toxicology. The use of Mn2/
as a probe for other divalent metal ions is evaluated and the enzymes and proteins containing
manganese are presented in an overview. The roles of manganese in plant lectins (concanavalin A),
phosphatases, xylose isomerase, and arginase are examined. Model complexes regarding redox
enzymes are elucidated, and individual accounts on manganese-containing catechol
dioxygenases, catalases, peroxidases, and superoxide dismutase, on the photosynthetic water
oxidation by the tyrosyl radical-manganese complex in photosystem II are reported.
With nearly 2400 references to assist further research, this book is an essential resource for
scientists and students in many disciplines, including bioinorganic, inorganic, and coordination
chemistry, bio-chemistry and-physics, molecular biology, enzymology, pharmacology, physiology,
clinical chemistry, nutrition,toxicology, and environmental sciences.
This book should facilitate the entry into and stimulate further research on the fascinating role of
manganese in biological systems.
167

				

